# Extreme erosion and bulking in a giant submarine gravity flow

**DOI:** 10.1126/sciadv.adp2584

**Published:** 2024-08-21

**Authors:** Christoph Böttner, Christopher J. Stevenson, Rebecca Englert, Mischa Schönke, Bruna T. Pandolpho, Jacob Geersen, Peter Feldens, Sebastian Krastel

**Affiliations:** ^1^ Institute of Geosciences, Kiel University, Otto-Hahn-Platz 1, Kiel, Germany.; ^2^ Department of Geoscience, Aarhus University, Høegh-Guldbergs Gade 2, Aarhus, Denmark.; ^3^ School of Earth, Ocean an Ecological Sciences, University of Liverpool, Liverpool, UK.; ^4^ Leibniz Institute for Baltic Sea Research Warnemuende, Seestraße 15, Rostock, Germany.; ^5^ GEOMAR Helmholtz Centre for Ocean Research, Wischhofstr. 1-3, Kiel, Germany.

## Abstract

Sediment gravity flows are ubiquitous agents of transport, erosion, and deposition across Earth’s surface, including terrestrial debris flows, snow avalanches, and submarine turbidity currents. Sediment gravity flows typically erode material along their path (bulking), which can dramatically increase their size, speed, and run-out distance. Hence, flow bulking is a first-order control on flow evolution and underpins predictive modeling approaches and geohazard assessments. Quantifying bulking in submarine systems is problematic because of their large-scale and inaccessible nature, complex stratigraphy, and poorly understood source areas. Here, we map the deposits and erosive destruction of a giant submarine gravity flow from source to sink. The small initial failure (~1.5 cubic kilometers) entrained over 100 times its starting volume, catastrophically evolving into a giant flow with a total volume of ~162 cubic kilometers and a run-out distance of ~2000 kilometers. Entrainment of mud was the critical fuel, which promoted run-away flow growth and extreme levels of erosion.

## Teaser

An initially small underwater avalanche entrained so much sediment along its path downslope that it grew over 100 times in size.

## INTRODUCTION

Sediment gravity flows are ubiquitous across Earth’s surface including terrestrial landslides, debris flows, and snow avalanches, as well as marine turbidity currents and debris flows. Entrainment of material into a gravity flow along its pathway (bulking) is a critical factor in how the flow behaves, governing flow concentration, rheology, speed, size, and ultimately run-out distance (*1*–*3*). Flow bulking is widely observed in small-volume terrestrial gravity flows such as snow avalanches (volumes of ~10^−4^ km^3^), which grow between 4 and 10 times the initial failure size (*4*), and debris flows (volumes up to ~10^−5^ km^3^) that have been reported growing up to 50 times larger than the initial failure (*5*). In submarine systems, flow bulking behavior has been conceptually described for decades via ignition theory (*1*). This framework predicts that flows will erode sediment along their pathway and grow larger and faster, which enables them to erode more sediment and run out farther. The positive feedback stops when sediment concentrations are high enough that viscous forces dominate, which suppresses turbulence and, thus, further erosion of the substrate (*1*).

It is well documented that submarine sediment gravity flows erode extensively along their pathway, which generates a variety of erosional channels and scour features hundreds to thousands of kilometers from source (*6*–*12*). However, it remains problematic to accurately map the depth, areal extent, and volume of sediment eroded by any individual gravity flow. This is because natural submarine systems are areally extensive, stratigraphically complex, and rather inaccessible (*13*). Only two submarine systems have flow bulking constrained by direct measurements: (i) The Bute Inlet, Canada, records relatively small-volume erosional bulking (0.5 × 10^−9^ km^3^) in gravity flows that grow over an order of magnitude and potentially up to 50 times in size (*14*), and (ii) the larger-scale Congo Canyon, offshore West Africa, documents one gravity flow entraining ~2.65 km^3^ of sediment along the channel floor, which resulted in flow self-acceleration and a large run-out distance of >1000 km (*15*). At a giant scale, substrate entrainment is also documented for the 1929 Grand Banks Event, offshore Newfoundland. This flow entrained between 50 and 100 km^3^ of sediment through just one of three canyon pathways it took down the continental slope, which equates to ~30 to 55% of its total deposit volume of 183 km^3^ (*16*). However, in all these cases, the volumes of the initial source failures are unknown or poorly constrained. Hence, it remains a fundamental problem to quantify and understand how much submarine gravity flows grow and evolve from initiation to final volume.

Here, we present a suite of acoustic data and sediment cores from the Agadir Canyon covering its upper catchment tributaries across the continental slope through to its distal reaches ~450 km downslope. From these data, we correlate the deposits of one of the largest gravity flow events on Earth (Bed 5, ~60 ka) (*10*), from its source in the canyon head through to the Canyon Mouth, and map its destructive, erosional pathway downslope. Our field data enable us to estimate the maximum initial failure volume within the canyon head (~1.5 km^3^) and compare this with the total deposit volume mapped beyond the Canyon Mouth (162 km^3^) (*17*). This stark mismatch in volume requires the flow to have entrained ~160 km^3^ of sediment through the Agadir Canyon and grown over 100 times its initial volume.

**Fig. 1.**
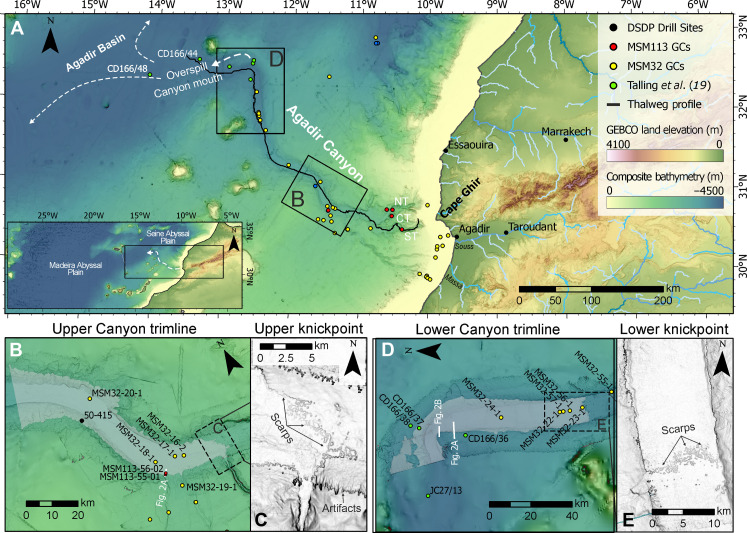
Overview map of the Northwest African margin showing the pathway of the Bed 5 event and its erosional marks on the seafloor. (**A**) Bathymetric map of the Agadir Canyon from the shelf edge down to the Canyon Mouth. Insert shows the wider Moroccan Turbidite System interconnected basins. DSDP, Deep Sea Drilling Project; GCs, Gravity Cores; GEBCO, The General Bathymetric Chart of the Oceans. Areal extent of erosion (gray overlay) across the Upper Canyon (**B**) and Lower Canyon (**D**). Slope maps (high/low slope = black/white color) detailing canyon floor knickpoint zones in the Upper Canyon (**C**) and Lower Canyon (**E**) with composite scours forming irregular (pocketed) erosional scarps (see fig. S1). Cores are marked with colored circles representing different research cruises. NT, Northern Tributary; ST, Southern Tributary; CT, Central Tributary.

The Agadir Canyon, offshore Morocco, Northwest Africa, is one of the largest submarine canyons in the World. It is ~450 km in length, up to 30 km in width, and 1.2 km in depth (Fig. 1). It is a conduit for some of the World’s largest submarine sediment-gravity flows that exceed 150 km^3^ in volume (*10*, *17*, *18*). The last giant flow to have passed through the Agadir Canyon was the “Bed 5 event,” which occurred ~59.4 ka between marine oxygen isotope stages 3 and 4 (*10*, *17*, *19*). The flow comprised ~162 km^3^ of sediment and had an exceptional run-out distance of >2000 km (*18*). The source area for the initial failure is interpreted to be from the upper reaches of the Agadir Canyon, along the Northwest Moroccan continental shelf (*18*). However, efforts to map and core these areas have found no coincident erosional hiatuses or notable landslide scars to explain the flow’s large volume (*20*, *21*).

## RESULTS

### General canyon morphology

**Fig. 2.**
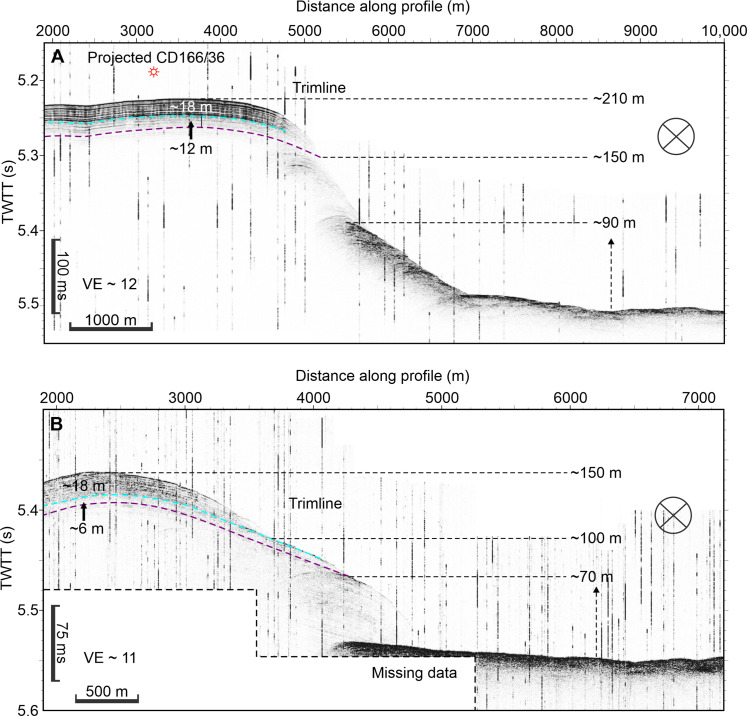
Profiles (3.5 kHz) across the erosional trimlines at various points along the canyon margins. (see Fig. 1) (**A**) Lower Canyon zone: a steep (and poorly resolved) step that cuts out 6 to 30 m of stratigraphy elevated between 90 and 150 m off the canyon floor. Note that core CD166/36 records Bed 5 without an erosional hiatus 210 m from the canyon floor. (**B**) Lower Canyon bend showing 6 to 24 m of erosion between 70 and 100 m above the canyon floor. TWTT, two‐way travel time; VE, vertical exaggeration.

We map the bathymetry of the Agadir Canyon from its head down to its mouth (Fig. 1). The canyon head zone comprises two deeply incised tributaries (northern and southern) with a less pronounced tributary situated between them (central). These tributaries cut back into the shelf edge and converge over ~80 km downslope into a single large conduit: the main Agadir Canyon (Fig. 1B). The gradient of the canyon progressively decreases from relatively steep values in the head region (~4° to 8°) to flatter slopes throughout the main canyon (~0.3°; Fig. 1A). Erosional trimlines manifest as distinct linear scarps between 10 and 30 m in height that run along the margins of the main canyon (Figs. 1, B to E, and 2 and see also fig. S2). These trimlines extend from prominent steep steps on the canyon floor called knickpoints, which are composed of numerous scours that variably amalgamate to form an irregular step between ~10 and 30 m in height in the Upper Canyon (Fig. 1C) and ~8 to 15 m in height in the Lower Canyon (Fig. 1E).Seismic profiles (3.5 kHz) intersecting the canyon margin scarps show that they are an expression of near-surface (i.e., relatively recent) erosion, which cuts out between 6 and 30 m into the underlying stratigraphy (Fig. 2). Mapping the trimlines reveals areally extensive (~4473 km^2^) major erosion along the entire length of the canyon (gray overlay in Fig. 1, B and D).

### Bed 5

**Fig. 3.**
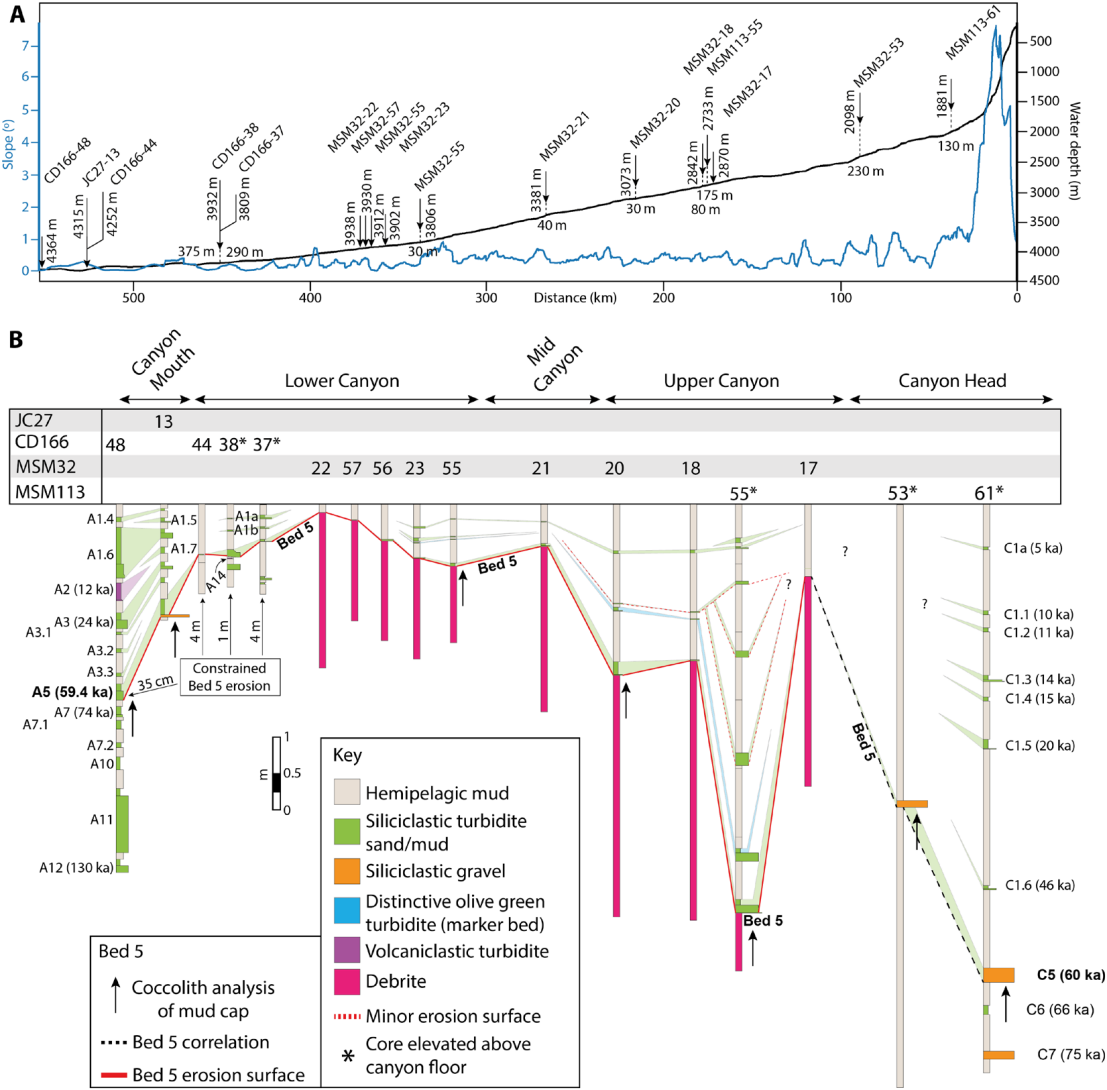
Core correlation along the length of Agadir Canyon following the Bed 5 event to its source. (**A**) Thalweg profile including bathymetric depth (black line) and slope gradient (blue line) from the Agadir Canyon head to its mouth (see Fig. 1 for profile position). Core locations marked with arrows with water depth (vertical number) and cruise code (45° labels). Several cores have elevated positions above the canyon floor (shown by dashed lines; a horizontal number gives height above the canyon thalweg). (**B**) Core correlation of Bed 5 and its erosion surface along the Agadir Canyon (A-coded beds within Agadir Basin and C-coded beds within the Agadir Canyon). Cores that are elevated above the canyon floor are highlighted with an asterisk. Note that core 61 ages on the basis of linear extrapolation from 72-ka coccolith biozone (see fig. S3).

**Fig. 4.**
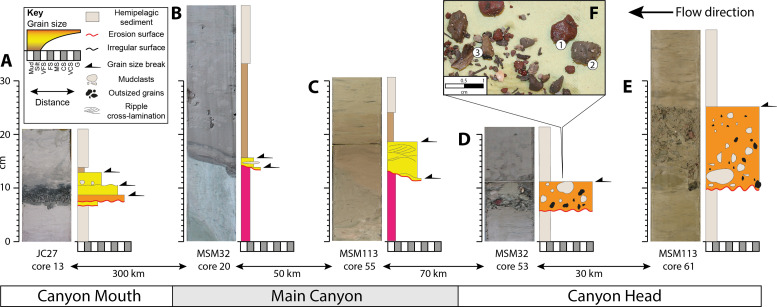
Examples of Bed 5 deposits correlated from the Northeast Agadir Basin (Mouth of the Agadir Canyon) up into the head of the Agadir Canyon. (see Figs. 1 and 5 for locations) (**A**) The Canyon Mouth records a thin gravel lag with basal erosion surfaces and several internal grain size breaks. (**B**) Along the canyon floor, it is characterized by a sharp basal erosion surface draped by very fine sand lag and a mud cap, which is often only a few centimeters in thickness. (**C**) Thirty meters above the canyon floor on the margins shows a steep erosion surface overlain by thicker ripple cross-laminated fine sand deposits. (**D**) Two hundred thirty meters above the canyon floor at the Tributary Confluence zone; Bed 5 is found as a thin gravel layer with large, outsized grains and mud clasts. (**E**) One hundred thirty meters above the Southern Tributary thalweg is a slightly thicker gravel layer with an erosive base highlighted by sheared mud clasts. (**F**) Bed 5 contains distinctive dark-red sandstone grains (1), armored mud clasts (2), some lithified mudstones (3), and a variety of shell fragments from the Moroccan Margin. Grain sizes: VFS represents very fine sand; FS, fine sand; MS, medium sand; CS, coarse sand; VCS, very coarse sand; G, gravel.

Deposits of Bed 5 are found in sediment cores, which are mapped using a robust correlation framework developed from the Agadir Basin (*10*, *17*, *19*, *22*) and extended upslope into the main Agadir Canyon and into the proximal Canyon Head region (Figs. 1 and 3A and see Methods). Age models were established in cores via a combination of radiocarbon dating of foraminifera and coccolith biostratigraphy (see Methods and fig. S3). Bed 5 is consistently recognized along the canyon at 60 ka with a distinct coccolith assemblage within its mud cap, which contains both old (451 to 443 ka) *Pseudoemiliania lacunosa* and young (291 ka to present) *Emiliania huxleyi* (fig. S4). It is the only gravity flow deposit in the stratigraphy to record this assemblage (*19*, *22*, *23*). Within the Agadir Basin (Canyon Mouth) region, Bed 5 is characterized by thin coarse-grained sand and gravel deposits overlying an erosive base (Fig. 4A). Within the Agadir Canyon, Bed 5 is represented by a widespread erosion surface that extends up the entire length of the canyon (Fig. 3B). The exact depth of erosion is not possible to constrain from the cores on the canyon floor because the substrate comprises a thickness of >50 m remobilized muds derived from the continental slope (*21*). However, cores situated high on the canyon margins show that in some places, Bed 5 was capable of eroding ~4 m in depth ~290 m above the Lower Canyon floor (CD166-37) (*19*). In the Agadir Canyon, the erosion surface is always draped by Bed 5 deposits, which comprise thin (in centimeters) sands across the canyon floor (Fig. 4B), while higher up on the canyon margins, they are thicker (tens of centimeters) sandy deposits (Fig. 4C). Across the Canyon Head region, Bed 5 is recognized in cores at the Tributary Confluence and in the upper reaches of the Southern Tributary (Figs. 3B and 4, D and E, and fig. S3). These cores record coarse gravel deposits at 230 and 130 m above the canyon floor, which contain distinctive red grains and old (>443 ka) armored mud clasts (Fig. 4E).

The sedimentary textures found in all the Bed 5 deposits indicate that the parent flow was extremely large and powerful, capable of suspending gravel >130 m above the canyon floor in the Southern Tributary (Fig. 4D), eroding along the entire 450 km in length of the canyon (Figs. 3 and 4), and ultimately bypassing almost all of its 162-km^3^ sediment load beyond the mouth of the canyon with a total run-out distance of ~2000 km (*10*, *17*, *19*). The grain sizes recorded in Bed 5 in the Canyon Mouth (Fig. 4A) and Canyon Head (Figs. 4, D and E) are used as a proxy to calculate minimum parent flow speeds (see Methods and table S1): core 61 in the Southern Tributary, ~4 m/s (up to 7 m/s) 130 m above the bed; core 53 at the Tributary Confluence, ~4 m/s (up to 7.7 m/s) 230 m above the bed; and core 13 in the Canyon Mouth, ~1.8 to 2.7 m/s (base of flow).

### Origins of Bed 5

**Fig. 5.**
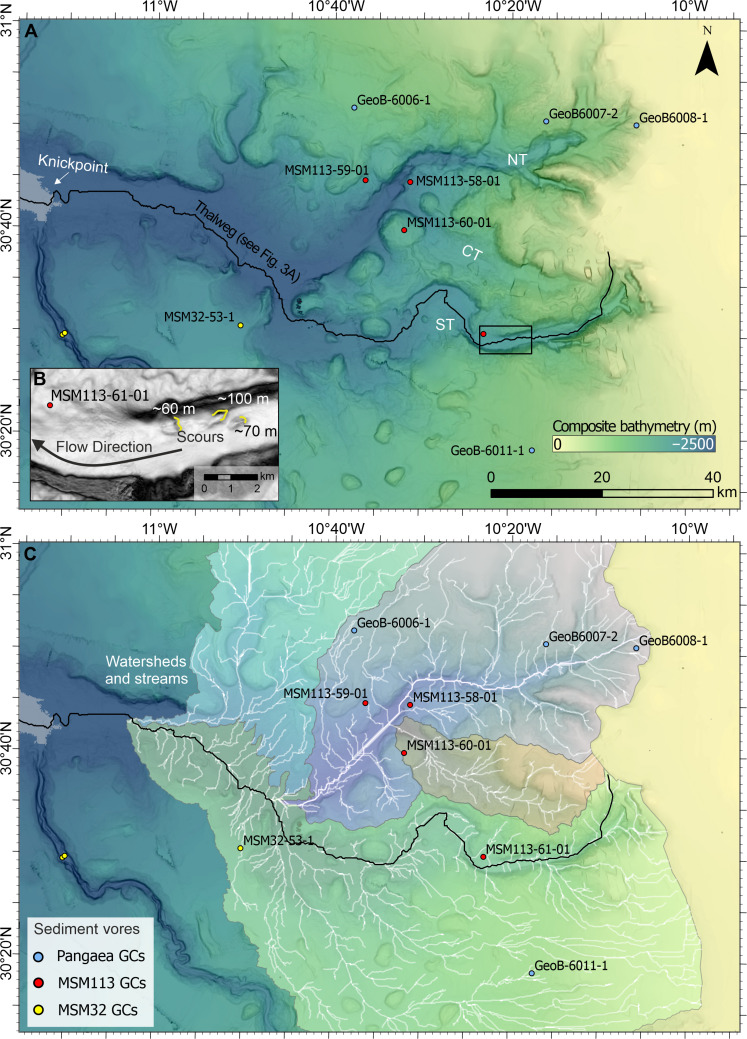
Bathymetric maps of the upper Agadir Canyon head zone highlighting the origin of the Bed 5 event. (**A**) Bathymetry of the Agadir Canyon head zone showing the Northern, Central, and Southern Tributaries. (**B**) Zoom-in gradient map of large scours seen on the floor of the Southern Tributary [location shown with box in (A)]. (**C**) Canyon head drainage patterns highlighting the Northern (purple), Central (orange), and Southern (green) Tributaries. Thalwegs are shown with white lines. Cores MSM113-58, MSM113-59, and MSM113-60, and GeoB6006, GeoB6007, and GeoB6008 rule out the Northern and Central Tributaries as Bed 5 pathways (see the main text for details).

We have correlated Bed 5 deposits into the upper reaches of the Southern Tributary (Fig. 3). Bed 5 deposits are coarse grained sand and gravel. The Southern Tributary shows a sandy canyon floor up to the shelf edge, which is surrounded by muddy sediments covering the continental slope (fig. S5). Therefore, it is likely that the source area for Bed 5 included a substantial amount of material remobilized from the Southern Tributary canyon floor and shelf-edge area. However, there is potential for Bed 5 to originate in multiple tributary catchments, which coalesce downstream into the main canyon system (Fig. 5). Cores located across the Canyon Head zone eliminate the other catchments as Bed 5 pathways. Within the Northern Tributary catchment (Fig. 5B), core GeoB6006 situated on the open slope records only hemipelagic mud down to ~100 ka (fig. S3). Cores MSM113-58 and MSM113-59 are positioned adjacent to the main Northern Tributary conduit and record hemipelagic muds down to ~82 ka (fig. S3). MSM113-58 is elevated ~270 m above the immediately adjacent canyon thalweg, which means that it could potentially miss a flow passing through the tributary. However, it sits on a bathymetric flat into which the Northern Tributary progressively incises, whereby upslope the elevation difference from flat-to-thalweg decreases to ~50 m. Any substantial flow event would overspill the upper parts of the Northern Tributary and spread across the flat. However, core 58 only captures a single gravity flow deposit comprising a 1-cm-thick very-fine sand dated at ~82 ka (fig. S3), which is too old to be Bed 5. Within the Central Tributary catchment, core MSM113-60 is situated on bathymetric high adjacent to its primary thalweg (Fig. 5B). This core is tentatively dated back to ~72 ka and records only one gravity flow deposit at 24 ka, which is too young to be Bed 5 (fig. S3). Flows passing through this catchment will also spread across the bathymetric flat sampled by core 58, before ultimately falling into the Northern Tributary (Fig. 5). A lack of gravity flow deposits of the correct age in both cores 58 and 60 indicates that Bed 5 did not pass through the Central Tributary. In summary, cores in both the Northern and Central Tributary show either no gravity flow deposits over the past ~85 kyr or isolated gravity flow deposits that are too young (24 ka; MSM113-60) or too old (82 ka, MSM113-58) to be Bed 5. This rules out the Northern and Central Tributaries as pathways for Bed 5, which leaves the Southern Tributary catchment as the only source area for the Bed 5 event.

### Mass balance

**Fig. 6.**
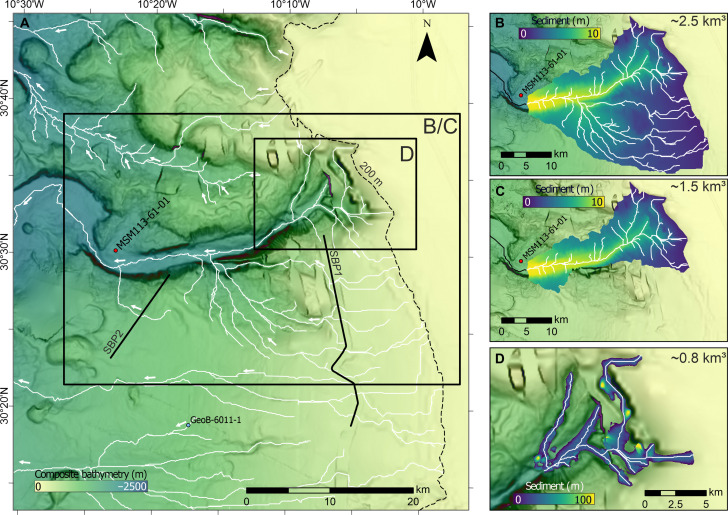
Bathymetric map of the Southern Tributary catchment with potential failure scenarios and associated volumes. (**A**) Bathymetry across the Southern Tributary catchment with thalweg networks mapped with white lines. The source area for Bed 5 must originate upstream of core MSM113-61, which records Bed 5 as a coarse-grained gravel deposit. Bed 5 slope failure scenarios are presented assuming total catchment failure (**B**), restricted catchment failure (**C**), and canyon-floor failure only (**D**). Initial failure volumes are calculated from the failure thickness and the areal extent of the failure (see the main text for details). The example in (D) uses 30-m average failure thickness.

On the basis of our new mapping, we can constrain the origin of the Bed 5 event to the upper reaches of the Southern Tributary (Figs. 3, 5, and 6). However, the volume of the initial source failure is difficult to estimate because no age equivalent or notable landslide scars can be identified within the tributary catchment (Fig. 6) (*21*). We address this problem by calculating volumes of two different failure scenarios: (i) assuming a catchment-wide failure that was thin-skinned enough to be below bathymetric resolution and (ii) assuming the failure occurred along the floor of the Southern Tributary, which left no diagnostic erosional features.

The first scenario assumes that the entire Southern Tributary catchment has suffered a thin-skinned failure, which is below the detection limit of the bathymetry (Fig. 6). To estimate a maximum potential initial failure volume, we assume that the thickness of the failure was equivalent to the resolution of the bathymetry at 0.5% of the water depth. This produces failure thicknesses of ~1 m in the uppermost tributary areas up to ~10 m in the deeper tributary thalwegs. The areal extent of the failure is mapped across the entire catchment, upstream of core MSM113-61 because it shows that the Bed 5 parent flow was already established and powerful enough to suspend gravel ~130 m above the canyon floor (Fig. 4E). This scenario results in an initial failure volume of ~2.5 km^3^ (Fig. 6B). However, shallow seismic profiles across the southern parts of the catchment are characterized by in situ layer-cake sediments indicative of undisturbed hemipelagic sedimentation (fig. S6). This rules out slope failure across about half of the southern catchment, resulting in an estimated failure volume of 1.5 km^3^ (Fig. 6C).

The second scenario assumes that the Bed 5 landslide scars are not detectable because they occurred as blanket remobilization of sediments along the floor of the Southern Tributary. This scenario is derived through insights from the contemporary Kaikōura Canyon, offshore New Zealand. A unique study by Mountjoy *et al.* (*24*) conducted repeat bathymetric surveys across the head of the Kaikōura Canyon, before and after a 7.8–*M*_w_ (moment magnitude) earthquake. This earthquake triggered substantial slope failures across the canyon head comprising ~1 km^3^ of material and produced a sediment gravity flow that ran out for >680 km. Critically, the majority of the failed sediment was sourced from the floor of the canyon, which suffered blanket remobilization between 10 and 20 m in depth (with localized zones up to 50 m) for ~15 km along the canyon thalweg (*24*). These slope failures did not leave obvious scars or trimlines through the canyon, and it is only due to the repeat surveys that the canyon floor failures were recognized. Using this analog, we estimate a range of potential volumes for the Bed 5 source failure by assuming an average thalweg failure thickness of between 10 and 50 m along the uppermost ~15 km of the Southern Tributary (Fig. 6D). This corresponds to canyon-floor failure volumes of between 0.3 and 1.5 km^3^ (fig. S7 and table S2). Canyon-floor failures thicker than 50 m are likely to generate erosional trimlines along the canyon margins that would be detectable in the bathymetry, particularly at the shallower water depths. As we do not see these features, we consider 50 m to be a reasonable upper limit of average canyon-floor failure thickness for the Southern Tributary.

The two scenarios produce similar upper estimates of the potential failure volume of ~1.5 km^3^. We use this upper estimate as a conservative measure of how much the flow must have grown from its initial failure into the giant Bed 5 event at the Canyon Mouth. Previously, Bed 5 deposits have been mapped across the wider Moroccan Turbidite System, which comprises a total deposit volume of 162 km^3^ (*17*, *18*). This is a stark difference of 160.5 km^3^ between the initial failure and total deposit volumes. Our data show that this “missing” sediment volume was incorporated into the flow through widespread erosion along the length of the Agadir Canyon (Figs. 1, B and D; 2; and 3B). This allowed Bed 5 to grow to at least ~107 times its initial size. The mapped trimlines show the areal extent of this major erosion surface totaling ~4,473 km^2^, which stretches almost the entire length of the canyon and hundreds of meters up the canyon walls (Fig. 1, B and D). To entrain ~160 km^3^ of sediment, the initial failure needed to erode to an average depth of ~35 m across this area. Our field data are in good agreement with this mass balance requirement showing 40- to 130-m-deep canyon-floor scours in the Southern Tributary (Fig. 5B), trimlines in the Lower Canyon cutting out ~24 to 32 m of stratigraphy 70 to 150 m above the canyon floor (Fig. 2, B and C), Lower Canyon cores showing ~4 m of erosion 290 m above the canyon floor (*19*), and, ultimately, Canyon Mouth cores constraining shallower erosion depths of between ~1 and 2 m (*22*, *25*).

## DISCUSSION

### Uncertainty in the initial failure volume

Our initial failure volume estimates are informed by the field data, which constrains the zone of failure to the Southern Tributary and restricted surrounding catchment. It also places upper limits on how thick the failures can be before they become visible on the bathymetry. It is possible that hemipelagic sediments have draped and buried the original landslide scar, which has obscured its detection on the bathymetry. Assuming an uninterrupted sedimentation rate of 10 cm/kyr in the upper tributary catchment over 60 kyr produces a hemipelagic thickness of 6 m (fig. S3). The resolution of bathymetry is 1 to 7 m in the upper catchment, which decreases with water depth to about 10 m along the canyon thalweg. Therefore, a 6-m-thick drape might obscure a scar <10 m in height in the upper catchment and <16 m in height in the deeper parts of the canyon thalweg. Buried scars of this size would still be seen on the 3.5-kHz profiles through onlapping reflectors. However, no buried scars are seen across the southern slopes (fig. S5), which constrains any buried failure to the canyon thalweg and adjacent catchment (Fig. 6C). Hence, a buried scar scenario has a relatively small potential volume (~0.5 km^3^), which is already accounted for in our estimates that explore a range of potential areal extents and failure thicknesses resulting in volumes from 0.3 to 1.5 km^3^ (Fig. 6 and table S2). The restricted areal extent of the initial failure means that to substantially increase this upper volume estimate requires much thicker

values of failure thickness (fig. S7), which are implausible because they would be detectable on the bathymetry. For example, an average failure thickness of 100 m within the Southern Tributary yields a volume of ~3.5 km^3^ (fig. S7). This would produce scarps and steps of ~100-m relief, which should be readily observable from the bathymetry with resolution between 1 and 10 m (shallow to deep; Fig. 6B).

Farther down the canyon erosional features are resolved at smaller scales, including localized scours of 60 to 100 m in depth (Fig. 5B) and trimline scarps of 8 to 24 m (Figs. 1 and 2). This supports the bathymetry interpretation that similar scale erosional features are not present in the Southern Tributary catchment, which points to a relatively thin-skinned failure. Therefore, we consider our initial failure volume of ~1.5 km^3^ to be a realistic upper limit. This means that the estimated flow bulking factor should be considered a minimum value, i.e., Bed 5 grew at least 100 times its initial size.

### Flow bulking behavior

Terrestrial debris flows typically have bulking factors around 2 to 4 (*2*, *5*), while snow avalanches generally grow up to around four to eight times their initial failure volume (*4*). The bulking factor documented for Bed 5 is over 100 and thus orders of magnitude larger. Direct measurements of small-volume submarine gravity flows in the Bute Inlet (10^−3^ to 10^−5^ km^3^) show similar bulking values, with an average 125 times increase in sediment discharge between moorings (*14*). In the larger-scale Congo Canyon, offshore West Africa, direct measurements and repeat bathymetric surveys show one gravity flow entraining ~2.65 km^3^ of sediment along the channel floor, which resulted in flow self-acceleration and a large run-out distance of >1000 km (*15*). While the initial failure volume is not known, the amount of eroded sediment is 31 to 91 times the annual discharge of the Congo River that feeds the submarine canyon system.

At a giant scale, only one example is appropriately documented for comparison: the 1929 Grand Banks Event, offshore Newfoundland. This submarine gravity flow entrained between 50 and 100 km^3^ of sediment through just one of the three canyon pathways it took down the continental slope (*16*). This equates to ~30 to 55% of its total deposit volume of 183 km^3^ found downslope across the Sohm Abyssal Plain (*26*). Conservatively assuming similar volumes of entrainment in each of the three canyon pathways, it is plausible that the 1929 Grand Banks Event could have entrained >150 km^3^ of sediment equating to >82% of its total deposit volume. Bed 5 shows similar volumes of sediment entrainment through the Agadir Canyon (~160 km^3^). However, the Grand Banks initial failure volume remains poorly constrained (*27*), which makes it difficult to assess how much the flow grew from initiation to its total deposit volume.

Extreme bulking appears to be a critical driver in the development of giant submarine gravity flows. However, the smaller volume systems described above also document bulking factors up to 125 times, particularly associated with the more powerful channel/canyon flushing flows. Hence, extreme bulking appears to be a generic propensity of submarine gravity flows and is a primary control on flow size, speed, and run-out distance.

### Bed 5 flow bulking

Why did Bed 5 grow so much and how was it able to entrain these vast amounts of sediment through the Agadir Canyon? Several factors will affect flow evolution: slope, initial flow speed, level of confinement, and the nature of the substrate along the pathway.

The slope of the Agadir Canyon comprises a steep canyon head zone between 1° and 4°, followed by the main canyon, which maintains a shallow gradient of ~0.3° for 350 km (Fig. 3A). Hence, the shallowing gradient is acting to slow the flow and cannot be the driver for downslope flow bulking.

Direct monitoring of contemporary canyons shows that gravity flows are sensitive to their initial speed, whereby flows exceeding 4 m/s are able to erode, maintain speed or self-accelerate, and develop large run-out distances (*15*, *27*). The grain sizes of deposits in the Southern Tributary (core 61) and Tributary Confluence (core 58) indicate that Bed 5 had minimum flow speeds of ~4.4 m/s 130 and 230 m above the canyon floor, respectively (see Methods). Given that the Bed 5 deposits sit high above the canyon floor, they likely represent the slower-moving upper parts of the parent flow (*28*). Direct measurements of smaller-scale submarine gravity flows show that velocity is highest close to the bed and at least three to five times faster than the upper parts of the flow (*29*–*31*). Therefore, it is reasonable to assume that Bed 5 frontal flow speeds along the canyon floor were substantially higher. Applying a three to five multiplier to our flow speed calculations from the Bed 5 deposits yields minimum frontal flow speeds on the canyon floor of between ~13 and 22 m/s in the Southern Tributary and Tributary Confluence. These speeds are comparable to the largest and fastest submarine gravity flows ever measured: the giant 1929 Grand Banks Event (19 m/s) (*26*), the 2006 Gaoping Canyon Event (20 m/s) (*32*), and rapid gravity flows related to the 2022 submarine Hunga-Tonga eruption (33 m/s) (*33*). Our flow speed estimates indicate that the Bed 5 event was well above the critical threshold of 4 m/s to bulk up and self-accelerate.

Canyon confinement is a function of flow volume and canyon cross-sectional area. Higher levels of confinement mean that flows become thicker for a given volume, which means that they travel faster and farther. The cross-sectional area of a canyon limits the amount of flow that can be confined such that as flows exceed a canyon’s cross-sectional area, their upper parts will overspill and rapidly deplete along the canyon margins. This means that flows will shrink to fit the size of the canyon they are passing through, reaching an equilibrium where they can maintain speed and run out for large distances. The cross-sectional area of the Main Agadir Canyon is massive (up to 36 km^2^). We note that the comparably sized 1929 Grand Banks gravity flow also passed through a canyon with an exceptionally large cross-sectional area (~20 km^2^) (*16*). These large canyon capacities are able to accommodate giant flow volumes (>100 km^3^) and allow flows to grow exceptionally large without suffering overspill and detrainment. The flip side of this canyon morphology is that smaller-volume flows will not fill up the large canyon and will be less confined for their size, i.e., smaller flows will have a thinner and wider cross-sectional profile compared to larger flows. This reduced confinement of smaller-volume flows means that they are more likely to quickly die-out along the pathway. This promotes a binary system whereby flows that are able to grow large enough become giant, while flows that are not able to bulk-up sufficiently quickly die-out within the canyon. This may explain the bimodal record of deposition downslope in the Agadir Basin, which records infrequent giant events with large run-outs or small-volume events that rapidly pinch out from the Canyon Mouth (*17*, *18*, *22*).

The erodibility of the underlying substrate and nature of the material along the flow pathway is critical to entrainment dynamics. In particular, entrainment of muddy sediments has been associated with flow bulking and extended run-outs in both terrestrial debris flows (*3*) and submarine gravity flows (*15*, *27*, *34*). The floor of the main Agadir Canyon is predominantly covered with thick (>50 m) deposits of remobilized mud, sourced from landslides on the Moroccan Continental Slope (*21*). The remobilization of these sediments destroyed the depth-related consolidation profile found in in situ seafloor sediments (*12*). Instead, the deposits produced thick accumulations of sediments with almost no strength changes with depth (fig. S8). Thus, Bed 5 was able to readily erode into this muddy substrate without becoming strength (i.e., depth) limited (*35*). Essentially, the flow was able to entrain as much muddy substrate as its capacity would allow.

### Mud and flow efficiency

The addition of mud (clay and silt) to a flow makes it highly efficient, greatly increasing run-out distance and enhancing transport of coarser sand-sized particles downslope (*8*, *36*, *37*). This is primarily due to mud having very low settling velocities, which means that it stays in suspension (i.e., maintaining flow density) for a long time, even at very low flow speeds. As mud is the finest grain size fraction within a flow and deposited last, its effects will apply to the flow’s entire life. In addition, muddy sediment is cohesive, which has a profound influence on flow rheology and carrying capacity. Experiments show that progressively increasing the proportion of clay in dilute flows results in an initial increase in turbulence and carrying capacity, followed by turbulence suppression due to the development of a cohesive laminar plug (*38*). This plug supports particles via yield strength, which potentially allows larger sand grains to be maintained in the flow below their critical shear stress (*39*). These effects make mud an excellent fuel to drive flow bulking, whereby entrainment of mud increases flow density and speed and then maintains those increases over the entire lifetime of the flow.

It is thought that flow bulking is limited by the flow’s sediment-carrying capacity, which increases with shear stress (flow speed) (*40*). As a flow entrains material, it will increase in size, concentration, and speed, which allows it to erode further. At some point, increasing sediment concentrations reach the limit of the flow’s capacity, which then suppresses further entrainment and stops flow growth (*1*). However, mud in suspension is extremely mobile, which means that a flow’s capacity to transport mud is very high (*41*). If entrainment of mud increases the flow’s bulk density and, in turn, speed (i.e., capacity), faster than it increases maximum sediment concentrations (limit of the flow’s capacity), then there is effectively no limit to flow bulking. This is a plausible mechanism to explain the genesis of Bed 5 with widespread erosion of mud along the entire length of the canyon and run-away flow growth. Bed 5 deposits located high above the canyon floor show that the parent flow completely filled and overspilled the canyon, which suggests that the canyon cross-sectional area was most likely the ultimate size limiting factor for the flow.

### Summary and implications

Giant submarine gravity flows can ignite from much smaller innocuous slope failures. Bed 5 originated as a small ~1.5-km^3^ canyon thalweg failure, which then eroded at least 160 km^3^ of sediment through the Agadir Canyon, growing >100 times its original failure volume and running out for over 2000 km. The extreme flow bulking was generated by run-away entrainment of mud along the floor of the Agadir Canyon. Mud is a critical fuel for substantial flow bulking because it makes flows highly efficient at transporting their sediment load. Once entrained, mud increases flow density, speed, and capacity and, due to very low settling velocities, maintains those effects over the lifespan of the flow. This effectively means that sustained entrainment of mud can drive indefinite flow growth, whereby the only limits on flow size are substrate availability and canyon morphology. Ultimately, the size of Bed 5 was only limited by the cross-sectional area of the canyon, which is exceptionally large and allowed the flow to grow into a catastrophic giant event. Extreme bulking may be a generic propensity of submarine gravity flows, with small and large volume systems all showing flow growth of an order of magnitude larger than seen in terrestrial systems. These insights highlight that the critical role bulking plays in submarine systems, which is primarily fueled by the entrainment of mud and ultimately governed by pathway morphology.

## METHODS

### Bathymetry and backscatter data

Bathymetry was collected aboard the RV Maria S. Merian on Cruise MSM32 between 25 September 2013 and 30 October 2013. The hull-mounted EM120 was operating at a nominal frequency of 12 kHz at a maximal swath width of 130° and is used for bathymetry and backscatter data. Processing included the application of sound velocity profiles, the application of manual and automatic methods to remove outliers, and the correction of angular dependence of the backscatter. Data were gridded to a resolution of 30 m using a Gaussian-weighted mean filter. Processing was done using the open-source software mbsystem (*42*) and commercial QPS software Qimera, Fledermaus, and FMGT.

### Geomorphologic analyses

All available spatial geoscientific information was imported into ArcGIS Pro (v3.1.3). Flow patterns were calculated using the basic hydrology workflow. All calculations and related graphs were done with Python. Relevant packages are Matplotlib (*43*), Pandas (*44*), and NumPy (*45*).

### Sediment echosounder data

The shallow subsurface was imaged using the hull-mounted parametric echosounder Parasound P70. Processing included a bandpass and envelope calculation. We used the IHS Kingdom software to visualize and interpret the acoustic data.

### Radiocarbon dating

The age-depth models of cores MSM113-58 and MSM113-61 are based on four accelerator mass spectrometry radiocarbon (AMS ^14^C) dates, of which three are of mixed planktonic foraminifera (predominantly *Globigerina bulloides*) and one is of pteropod shells (fig. S3). AMS ^14^C dating was performed at the Beta Analytic Testing Laboratory, Miami, FL, USA. Radiocarbon ages were calibrated using Calib8.2 (*46*) the Marine20 calibration dataset (*47*). There is no information about the local marine reservoir correction (Δ*R*) for the Agadir Canyon.

### Coccolith biostratigraphy

Coccolith biostratigraphy was established by counting the abundance of five key species within hemipelagic sediments [(*22*) and references therein]: *P. lacunosa*, *Gephyrocapsa caribbeanica*, *Gephyrocapsa aperta*, *Gephyrocapsa mullerae*, and *E. huxleyi*. We identified hemipelagic sediment by a lack of sedimentary structures, randomly dispersed foraminifera, fine-grained texture (clay and silt), and abundant bioturbation, which produces a mottled gray/brown coloration. Smear slides were prepared by smearing a toothpick head of sediment onto a glass slide, which was then mixed with a drop of distilled water, dried on a hot plate, and then covered slide fixed with an appropriate glue. Over 300 coccoliths were counted per slide under a transmitted light microscope at ×1600 magnification. Only the *G. mullerae/E. huxleyi* biozone (72 ka ± 5 kyr) was identified in the cores. Coccolith assemblages within gravity flow deposits were also analyzed via the same approach. This provided insights into the source material’s age and/or depth of erosion by the parent flow (fig. S4).

### Core descriptions

All cores were visually logged at 1:4 scale, which identified hemipelagic sediments (mottled gray/brown muds with scattered foraminifera) and gravity flow deposits (sharp/erosional bases, green/brown-colored sand to gravel grain sizes, occasional sedimentary structures such as planar lamination and ripple cross-lamination, fragments of shells, and often graded or show stepped fining upward trends).

### Core correlation

Bed 5 is correlated between cores using a robust core correlation framework well established in the Agadir Basin and mouth of the Agadir Canyon (*10*, *17*, *19*, *22*, *23*, *48*). Bed 5 is identified using several lines of evidence: (i) Age of emplacement is estimated at ~60 ka (between Marine Oxygen Isotope Stages 3 and 4) with the age model for cores established through carbon dating of planktic foraminifera, pteropod shells, and identification of the *E. huxleyi/G. mullerae* transition zone (72 ka) and sedimentation rates extrapolated from nearby dated GeoB cores (Fig. 5A and fig. S3); (ii) The composition of Bed 5 deposits is siliclastic (Moroccan Margin source) with a dominance of quartz and the presence of distinctive dark-red sandstone grains; (iii) The coccolith assemblages within Bed 5 deposits are distinct from similar aged events found across the Moroccan Turbidite System (fig. S4); and (iv) Bed 5 is unusually coarse grained and large volume with its deposits characterized by gravel lags or mud-draped erosion surfaces. In the past 200 kyr, no event has produced deposits coarser than fine sand (*17*).

### Initial failure volume estimate

We performed all bathymetry-based work and volumetric calculations in ArcGIS Pro (version 3.1.3). The drainage pathways, water shed areas, and talweg profiles of the canyon head were calculated using the basic hydrology workflow. We used the shelf break (200-m isobath) as the upslope limit and the confluence of all head tributaries as the downslope limit (see Fig. 5A).

To calculate the first scenario, we calculated the vertical resolution of our multibeam data (0.5% of the water depth) by simple raster multiplication. We used ArcGIS Pro’s watershed function to outline the two drainage areas (two separate areas) that contribute to the sediments of core MSM113-61 (showing Bed 5). As a final step, we masked the vertical resolution raster with the two drainage areas and calculated the volume of this grid with ArcGIS Pro’s surface volume function.

To calculate the second scenario with a blanket erosion, we used the Kaikōura Canyon for comparison (*24*). To estimate the volume of a blanket erosion of 30 m, we created points every hundred meters along the thalweg profiles of the upper canyon, cut them at the 200-m isobath, extracted the elevation at those points, and added 30 m to the extracted values (10, 20, 50, and 100 m are shown in fig. S7). We used the Empirical Bayesian Kriging (power semivariogram) with a 1000-m search radius and 0.2 smoothing factor (smooth circular neighborhood) to extrapolate these values across the canyon floor. We subtracted the resulting grid from the original bathymetry. As a final step, we calculated the volume of this difference grid with ArcGIS Pro’s surface volume function.

### Reconstructing flow properties

#### Grain size

The settling velocity of a particle can be related to the lateral flow speed needed to suspend it via (*49*)

$$w_{s}=\frac{v}{d}\;\left[{\left(10.36^{2}+1.049D_{*}^{3}\right)}^{\frac{1}{2}}-10.36\right]$$ (1)

where *v* is the kinematic viscosity of water at 10°C and a salinity of 35 parts per thousand (1.36 × 10^−6^ m^2^/s), *d* is the diameter of the particle (table S1), and D_*_ is the dimensionless grain size via

$$D_{*}={\left[\frac{g\left(s-1\right)}{v^{2}}\right]}^{1/3}d$$ (2)

where *g* is the gravitation acceleration (9.81 m/s^2^) and *s* is the ratio of densities of particle and water (2650/1027 kg/m^3^ = 2.58). Note that this approach also assumes dilute flow conditions and that, with higher concentrations, hindered settling will reduce settling velocities and, in turn, produce slower estimates of flow speed. However, it is not possible to assess the contribution of hindered settling in this instance because we do not know the flow concentration at various points along the boundary layer (i.e., canyon floor and high up on the margins). Hence, Eq. 1 should be considered a first order estimate of flow speed.

## Supplementary Materials

### This PDF file includes:

Supplementary Text Figs. S1 to S8 Tables S1 and S2 References
