# Supplementary material for: Extreme erosion and bulking in a giant submarine gravity flow
Source: Sci Adv. 2024 Aug 21;10(34):eadp2584. doi: 10.1126/sciadv.adp2584 (PMC11338220; doi:10.1126/sciadv.adp2584)
Supplement: Supplementary file 1 — Supplementary Text Figs. S1 to S8 Tables S1 and S2 References [file sciadv.adp2584_sm.pdf]

Science Advances
AAAS

Supplementary Materials for

Extreme erosion and bulking in a giant submarine gravity flow

Christoph Böttner *et al.*

Corresponding author: Christoph Böttner, christoph.boettner@geo.au.dk;
Christopher J. Stevenson, chriss@liverpool.ac.uk

Sci. Adv. **10**, eadp2584 (2024)
DOI: 10.1126/sciadv.adp2584

This PDF file includes:

Supplementary Text
Figs. S1 to S8
Tables S1 and S2
References

Knickpoint Profiles

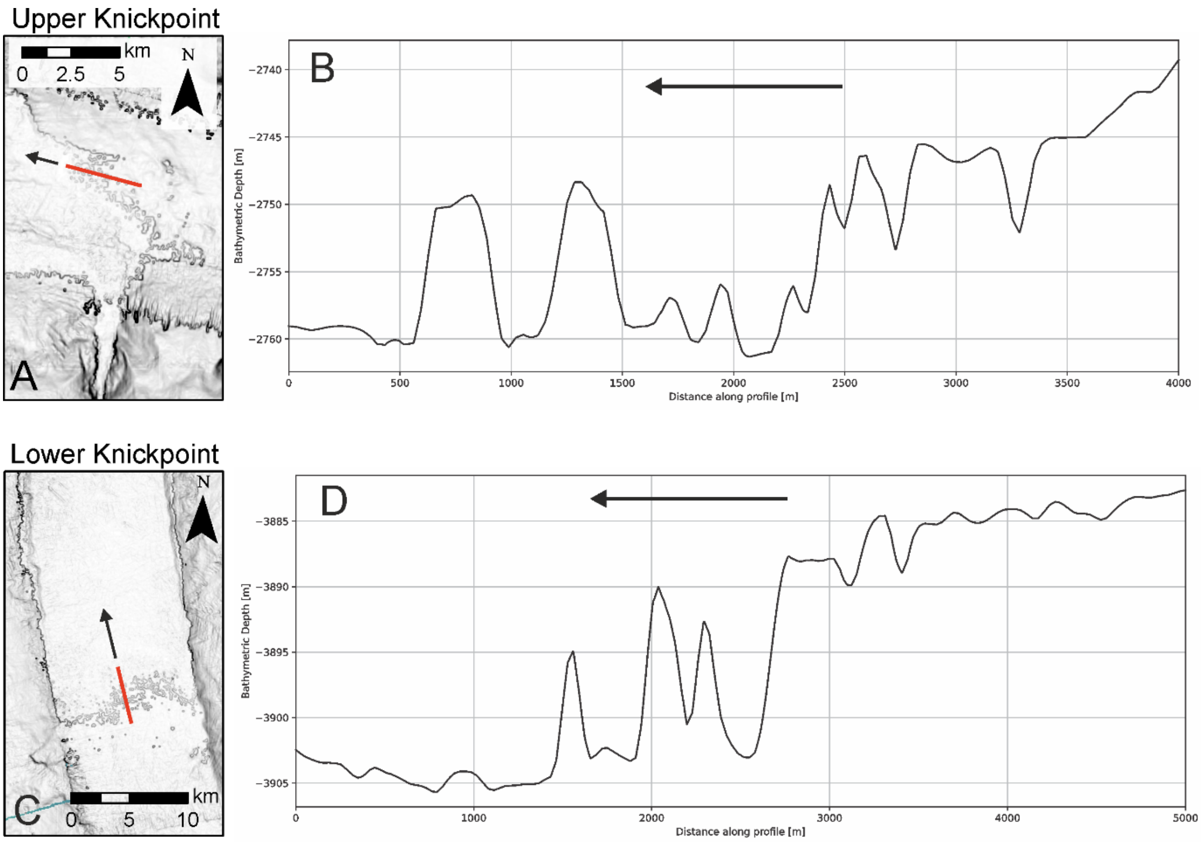

Figure S1. Bathymetric slope maps of the Agadir Canyon's upper and lower knickpoints*. Black is high slope gradient; white is low slope gradient. (A) Slope Gradient map of the upper knickpoint with indicated depth profile (red line) shown in (B). (C) Slope Gradient map of lower Knickpoint with indicated profile (red line) shown in (D). Location of A and C are given in Figure 1.*

Trimline & Knickpoint

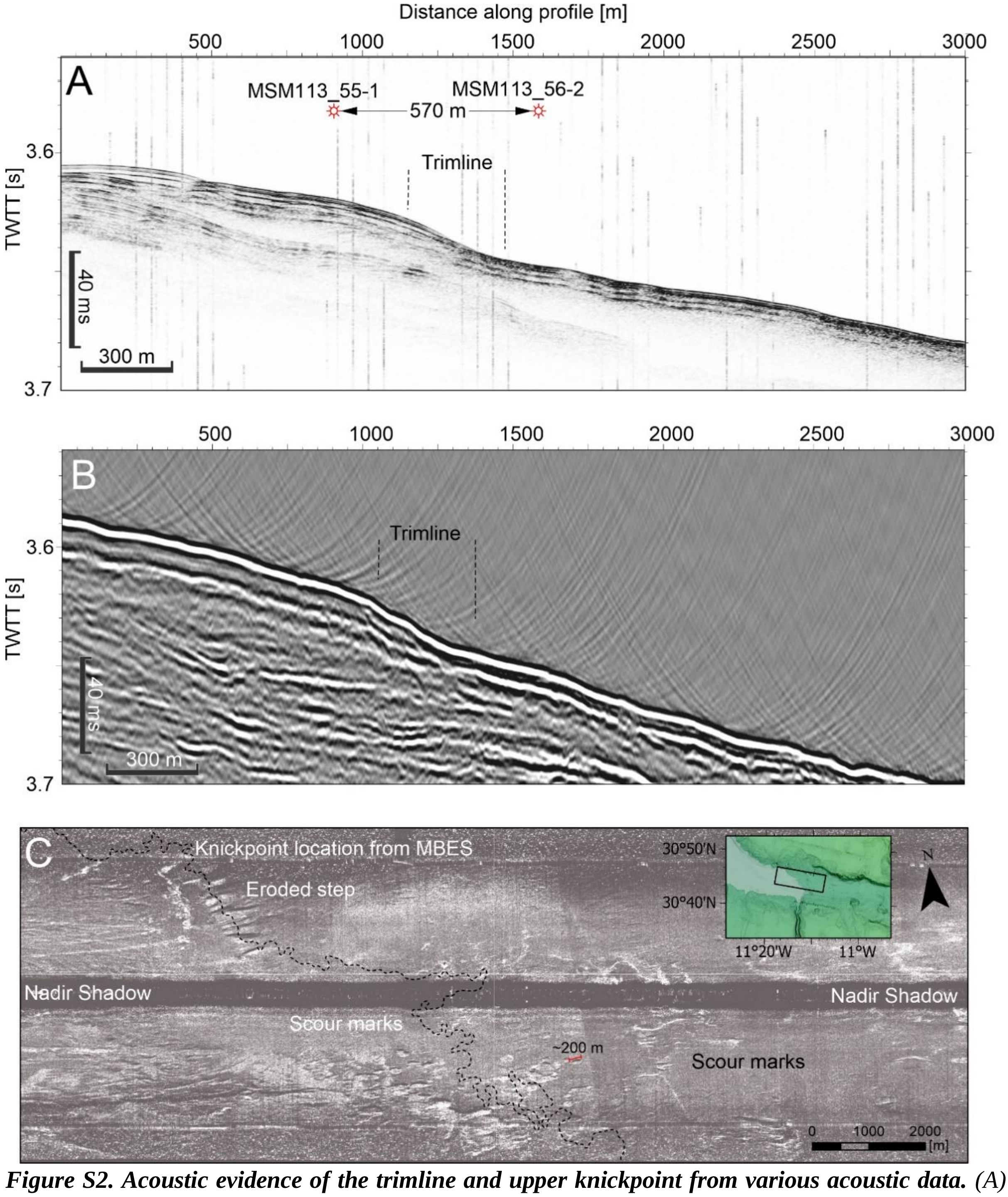

Figure S2. Acoustic evidence of the trimline and upper knickpoint from various acoustic data. *(A) 3.5 kHz parametric echosounder data across the trimline in the upper canyon. The profile covers the locations of cores MSM113-55-1 and MSM113-56-1 (see Fig. 1 for position). (B) High-resolution seismic image across the same location as in (A), showing the trimline is also visible in seismic data despite being close to the resolution limit (~5 m vertical resolution @ 80 Hz, 1500 m/s). (C) Side-Scan Sonar data showing detailed morphology of the upper knickpoint with numerous erosional scours (up to 200 m long) forming a composite scarp (~60 m high). Note the highly irregular crestline of the scarp and the large V-shaped cut, which incises upslope from the main scarp crestline by 1-2 km.*

Seismic data

2D high-resolution seismic data were recorded using an 88-channel Geometrics GeoEel streamer with a standard GI-gun (1.7L) as source. Processing was done with Vista Seismic Data Processing Software and included trace binning, filtering, NMO-correction, stacking, and post-stack finite-difference migration. The data has a vertical resolution of ~ 4.5 m (at 80 Hz and 1500 m/s seismic velocity) and a bin size of 2 m (see *Figure S2B*). The IHS Kingdom software was used to visualize and interpret the seismic data.

Sidescan sonar

Towed Ocean Bottom Instrument (TOBI) is an instrumented vehicle, which is towed close to the bottom of the deep ocean from a ship and uses sound to form detailed images of the sea floor. The TOBI system deploys a deep towed dual sidescan sonar system based around 30 KHz. The range to each side of the nadir (central zone) is 3 km, yielding a total swath width of 6 km. The seabed footprint ranges from about 4 x 7 m close to the vehicle track to 42 x 2 m at far range (see *Figure S2C*).

Bed 5 grain sizes

**Core**	**D_{50} (mm)**	**D_{90} (mm)**	**D_{max} (mm)**
MSM113-61	1	2	12
MSM32-53	1	2	15
JC27-13	0.5	1	2

Table S1: Grain size information for Bed 5 deposits used to reconstruct parent flow speeds *(see Methods). Note in the Canyon Head region deposits comprise a coarse-grained matrix with occasional much larger outsized particles. Hence, the large difference between D_{50}, D_{90} and D_{max}. Speed estimates from grain size use D_{90} and the D_{max} values.*

Core Age Models

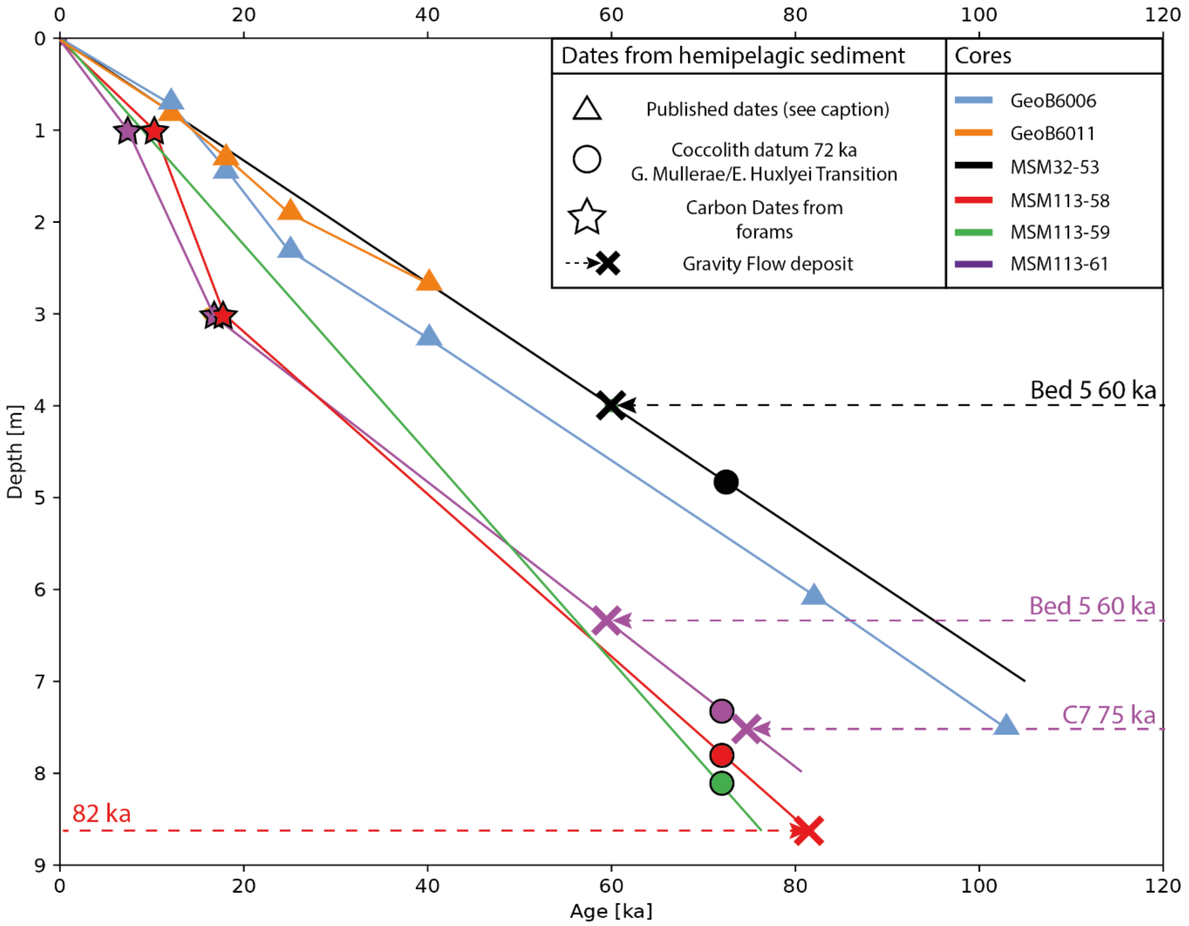

Figure S3. Age models for cores across the Canyon Head region *(Fig. 5), which include GeoB cores situated on the open slope ~10 km away from the tributary thalwegs (after 50). MSM cores dated in this study show similar age trajectories to the GeoB records: MSM32-53 showing a sedimentation rate of ~7 cm/kyr, and MSM113-58, 59 and 61 having slightly higher sedimentation rates of ~10 cm/kyr. Note that Core 59 does not have carbon dates meaning the linear projection likely misses the stepped profile seen in the upper parts of 58 and 61. The error from the E.Huxleyi/G. Mullerae coccolith biozone (72 ka) is ±5000 years. The position of select gravity flow deposits are shown with dashed arrows and an X with corresponding age of emplacement. These deposits are shown as they are potential candidates for Bed 5. Bed 5 is identified in Core 53 as the only gravity flow deposit in the core (dated at ~60 ka). MSM113-61 has two potential gravity flow deposits at 6.2 and 7.3 m depth. The 72 ka coccolith biozone occurs at ~7m, which constrains the ages of these deposits (Bed 5 at 60 ka and C7 at ~75 ka). Note that C7 occurs below the coccolith biozone and must be older than 72 kyr. MSM113-58 only records one gravity flow deposit below the 72 ka coccolith biozone (dated at ~82 ka), which rules it out as being Bed 5.*

Core MSM113-60

Core 60 records 7.8 m of hemipelagic sediment and one gravity flow deposit at 2.6 m depth. The base of Core 60 potentially captures the 72 ka coccolith biozone with appropriate ratios of G.M/E.H species. However, slightly deeper penetration is needed to confirm the upward trend in species, i.e. to see the transition. Without the longer record, it is difficult to confirm the presence of the 72 ka biozone. As an estimate, we tentatively interpret the coccolith ratios found at the base of Core 60 at 7.88 m hemipelagic depth as the 72 ka biozone. This gives an average sedimentation rate of ~11 cm/kyr, which is similar to

nearby Cores 58, 59 and 61. With this age model, the gravity flow deposit at 2.6 m is ~24 ka, which is too young to be Bed 5.

Coccolith assemblages of Bed 5

The coccolith assemblages within Bed 5 are distinct from surrounding event beds (*22*). They contain both *E.Huxlyei* (young) and a small proportion of *P.Lacunosa* (old). This mixture of species indicates that the Bed 5 event was sourced from a failure at least 35 m thick (assuming uninterrupted hemipelagic sedimentation between 450 ka and 60 ka) or the parent flow was able to erode down up to 35 m along its pathway. Canyon floor scours up to 100 m deep in the Southern Tributary suggest that flows were powerful enough to erode substantially into the canyon floor. However, it is not possible to determine how much of that scouring was produced by Bed 5 alone.

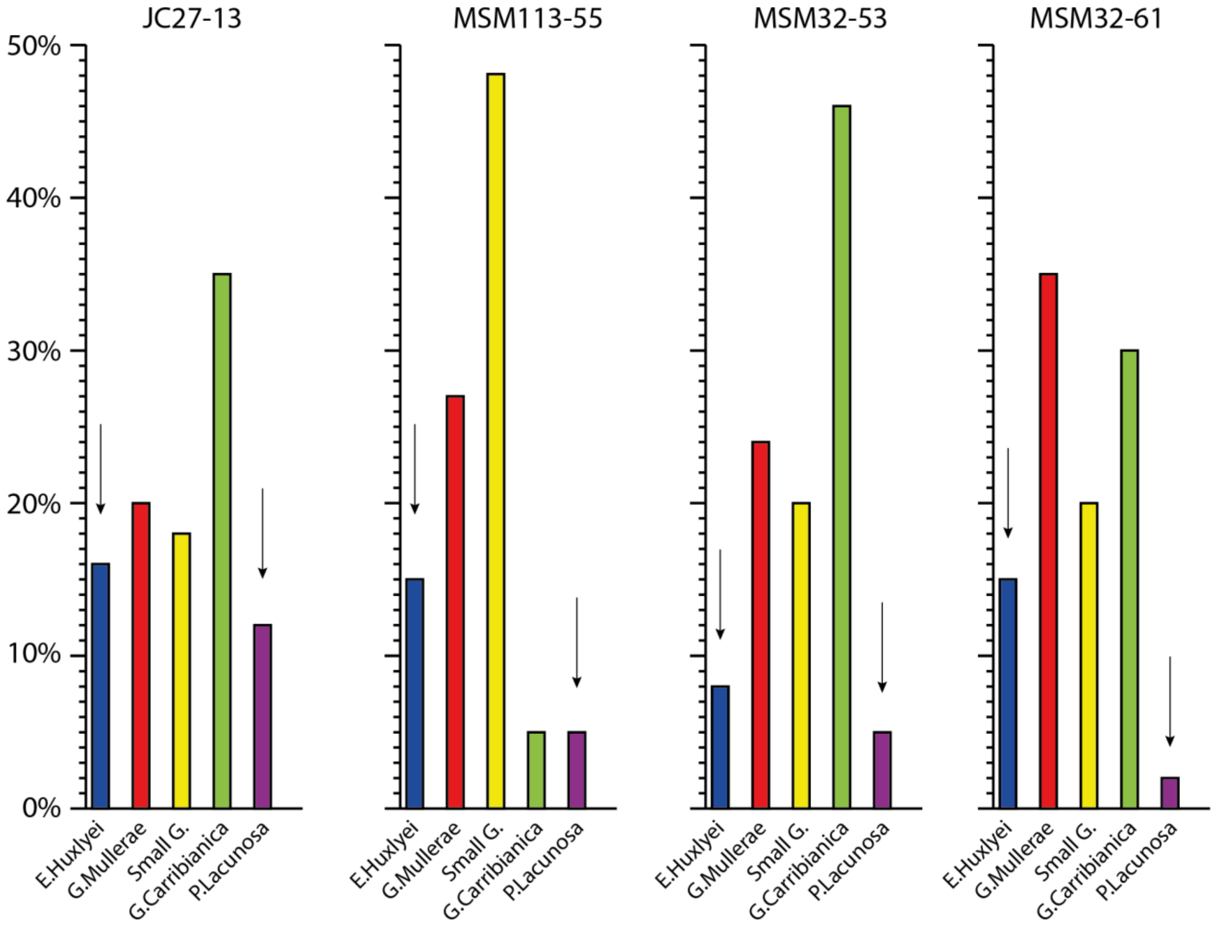

Figure S4. Coccolith assemblages from the mud cap of Bed 5 for select cores from the Canyon Mouth (JC27-13; After 22), Main Canyon (MSM113-55), Tributary Confluence (MSM32-53) and Southern Tributary (MSM113-61). *Bed 5 coccolith assemblages are distinct from other events because they characteristically contain both E.Huxlyei (8-15%) and a limited amount of much older P.Lacunosa (2-12%). The intervening Gephyrocapsa assemblages typically show a substantial proportion of G.Mullerae (~20-30%), Small G. (~20%) and a dominance of G.Carribianica (30-48%). Core 55 is different with only 5% G.Carribianica and a dominance of Small G. (48%). However, it still records the diagnostic species of E.Huxlyei and P.Lacunosa. No other events younger or older than Bed 5 capture both E.Huxlyei and P.Lacunosa (see 22).*

The canyon erosion surface

The widespread erosion surface recorded in all cores in the Agadir Canyon is interpreted to have been generated by the Bed 5 Event. Two lines of evidence support this interpretation. First, Bed 5 is the only large-volume event to record substantial erosion in the Lower Agadir Canyon and Agadir Basin over the past 200 kyr (Fig. 3B; 19, *22*). Second, the erosion surface throughout the canyon is always draped by Bed 5 deposits (identified by age, composition and coccolith assemblages), even in cores elevated above the canyon floor (Fig. 3B; Core 55). This indicates a close temporal relationship and likely genetic link between the canyon erosion and subsequent emplacement of Bed 5 ~60 ka.

An alternative interpretation is that Bed 5 passively drapes an older erosion surface, hence is not genetically related to the incision. If this were the case, we would expect to record a variety of stratigraphy >60 ka between the erosion surface and Bed 5, particularly in cores on the canyon margins that are more likely to preserve hemipelagic sediments between event beds (e.g. Core 55). However, Bed 5 always immediately overlies the erosion surface in any position within the canyon (see also *19* for canyon margin cores across the lower canyon). Hence, we interpret the erosion surface as being generated by the Bed 5 Event.

Initial failure volume estimates

Whilst the initial failure for Bed 5 is not visible, the field data does rule-out zones of the Southern Tributary catchment as source areas and highlights the Southern Tributary thalweg as a local source of coarse-grained sediment. Figure S5 interprets a shallow-seismic profile across the upper slope, which shows no widespread slope failures.

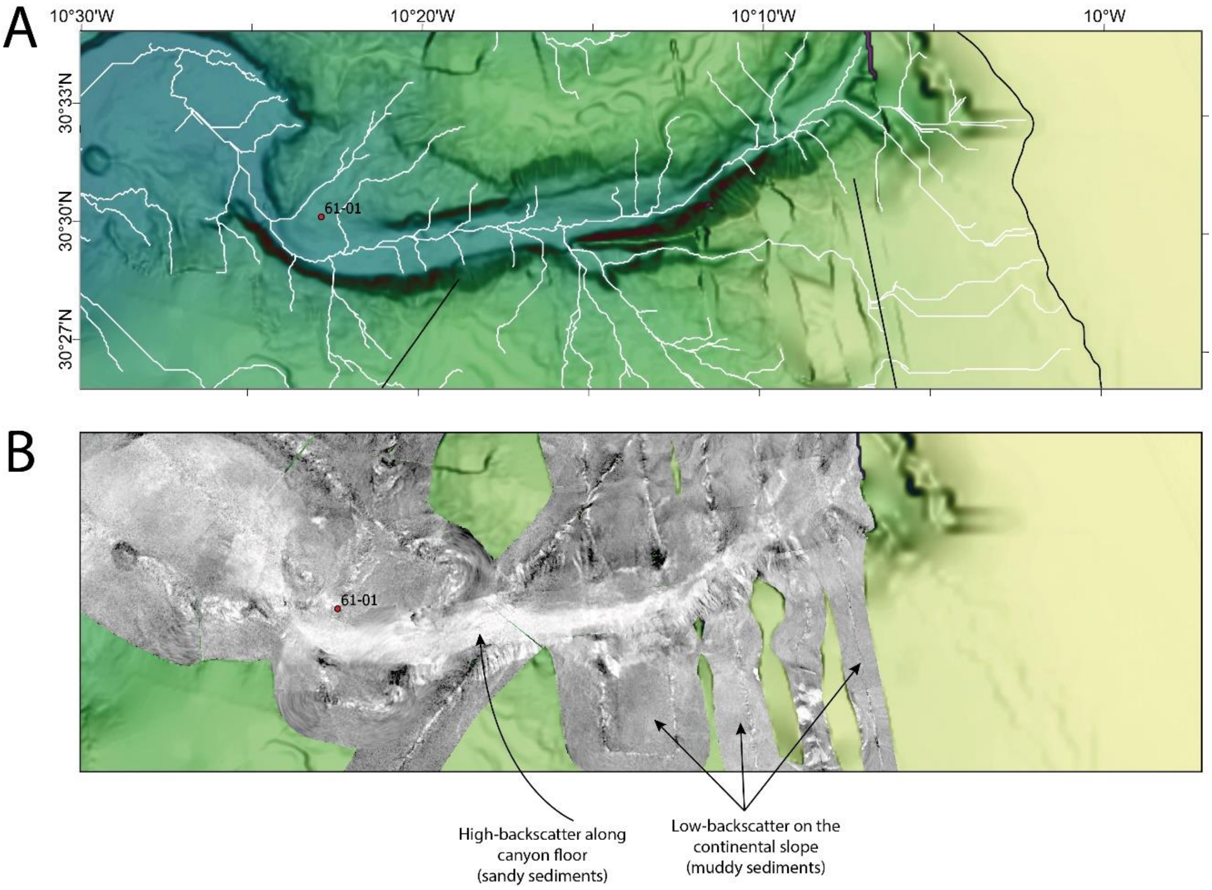

Figure S5. Multibeam bathymetric and backscatter data from the upper Southern Tributary. *(A) Swath bathymetry across the upper Southern Tributary thalweg and adjacent catchment. Drainage thalwegs shown as white lines. Core MSM113-61 (Core 61) shown as a red circle. (B) Backscatter image from the swath bathymetry in (A). Note the Southern Tributary canyon floor has a high-backscatter response, indicating a covering of sandy sediments. In contrast, the adjacent continental slopes have a low-backscatter response, which indicates mud-dominated strata (see also Fig. S6 below). As Bed 5 contains a substantial proportion of coarse-grained sand and gravel (e.g. Core 61), it is likely the initial failure included sediments from the canyon floor.*

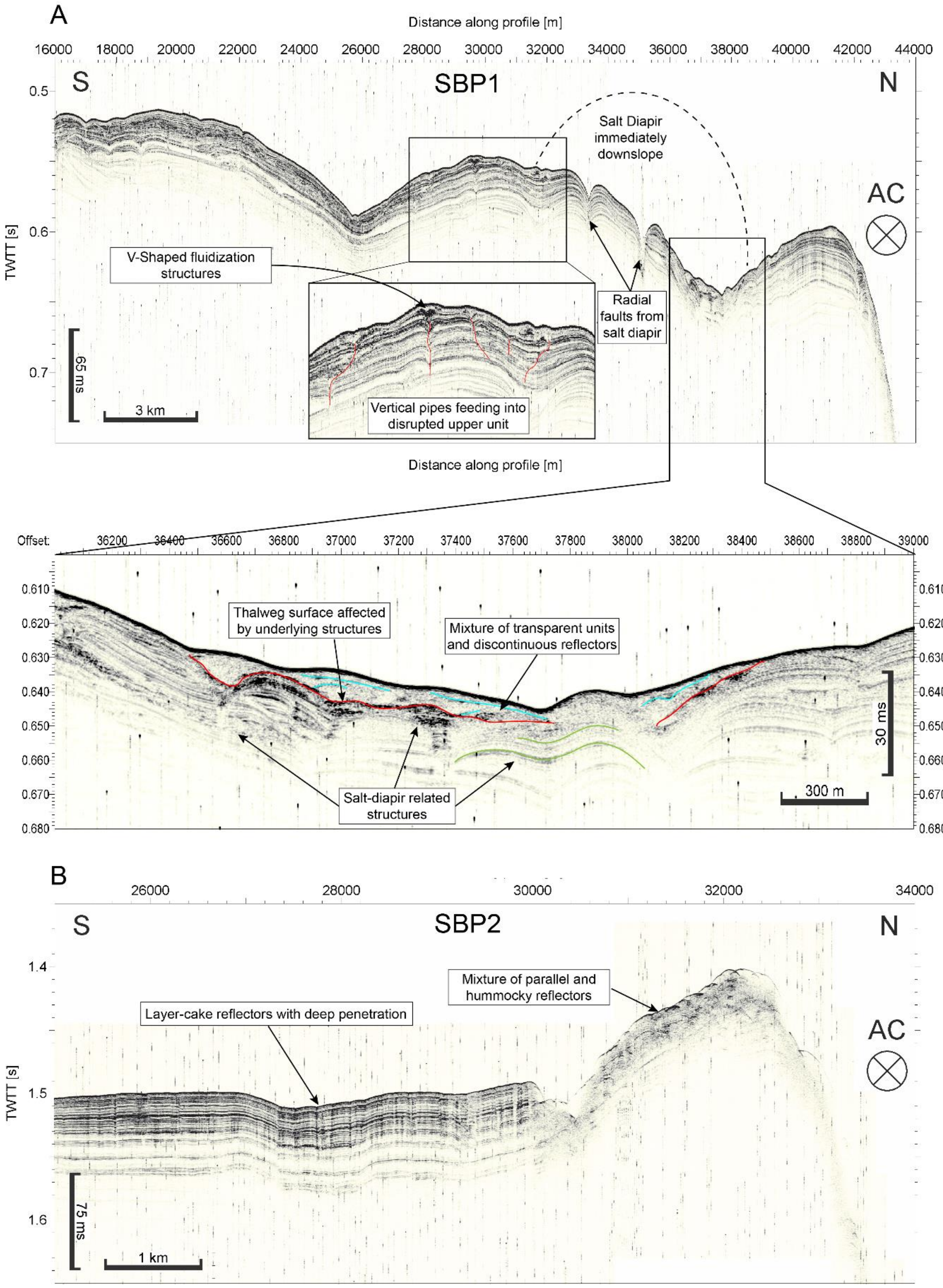

Figure S6. Sub-bottom profiles across the southern parts of the Southern Tributary catchment *(see Fig. 6A for locations). (A) SBP1 covers the upper slope and is characterized by flat parallel reflectors with deep penetration. These acoustic facies indicate in-situ (undisturbed) hemipelagic muds and rule-out widespread slope failure across this part of the catchment. Note two zones of disturbed sediments: a weakly remobilized unit in the central part of the profile, and a locally thick transparent unit sitting within a minor thalweg on the northern part of the profile. The weakly remobilized unit is subtended by several vertical pipes, each of which are loci for V-shaped deformation (see zoom in). This style of deformation is characteristic of fluidization: injection structures from either water or gas escape (e.g.*

51, 52). For clarity the transparent unit sitting within the thalweg is interpreted as a separate zoom image. The unit actually comprises several transparent units with weakly developed, discontinuous flat reflectors that variably down- and onlap onto the transparent units. The basal erosion surface mimics underlying structural features and shows syn-sedimentary relationships at depth (below the transparent unit). Hence, this transparent unit and the erosion surface it sits on most likely represent a series of local slope failures, which are related to some structural control. The most likely structural control is an adjacent (immediately downstream) salt diapir, which diverts the thalweg and produces radial faults that are seen on the SBP1 profile. The salt diapirism is also a likely driver of fluid migration, and in turn, a control on the fluidization structures to the south. (B) SBP2 profile shows flat parallel reflectors, indicating in-situ hemipelagic muds. This rules-out widespread slope failure across this area of the catchment. Note the mixed acoustic facies adjacent to the Southern Tributary, which shows hummocky transparent units variably interleaved with flat reflectors. This is interpreted as being the products of a series of locally restricted, thin-skinned slope failures.

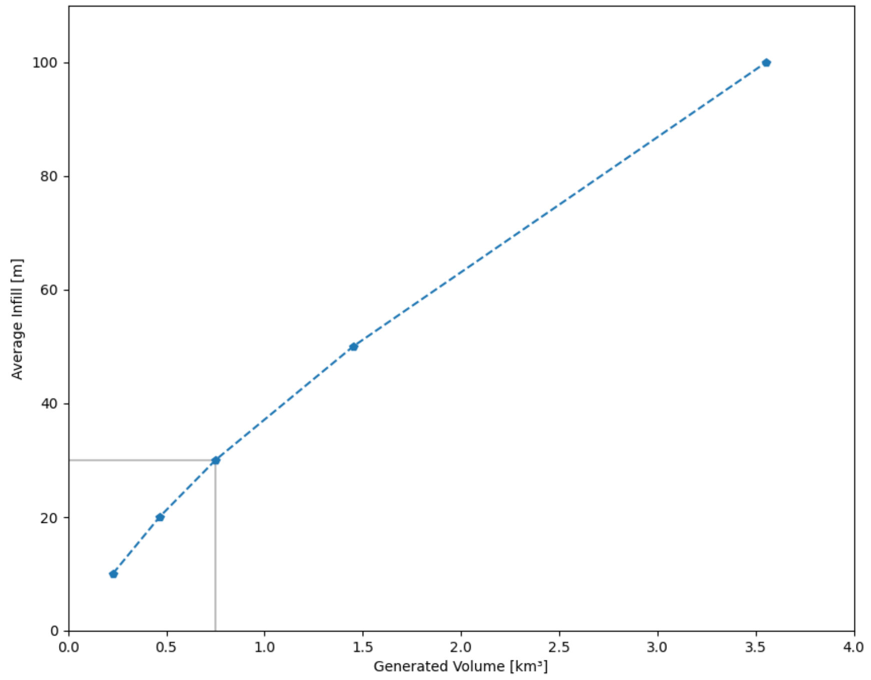

Figure S7. Plot showing the relationship between thickness of the initial thalweg failure and the resultant volume generated by the failure *(Fig. 6D for area). Table S2 below provides some scenarios.*

Average thickness of failure (m)	Volume of sediment failed (km^3)	Bulking factor (162 km^3/initial failure volume)
10	0.2926	553
20	0.5404	299
30	0.833	193
50	1.525	105

Table S2. Showing a range in the average thickness of thalweg failure in the Southern Tributary, and how this corresponds to variations in the estimated initial failure volume. *Note that the range in estimated volumes (0.3-1.5 km^3) is relatively minor compared to the total deposit volume down slope of 162 km^3.*

Shear Strength Profiles

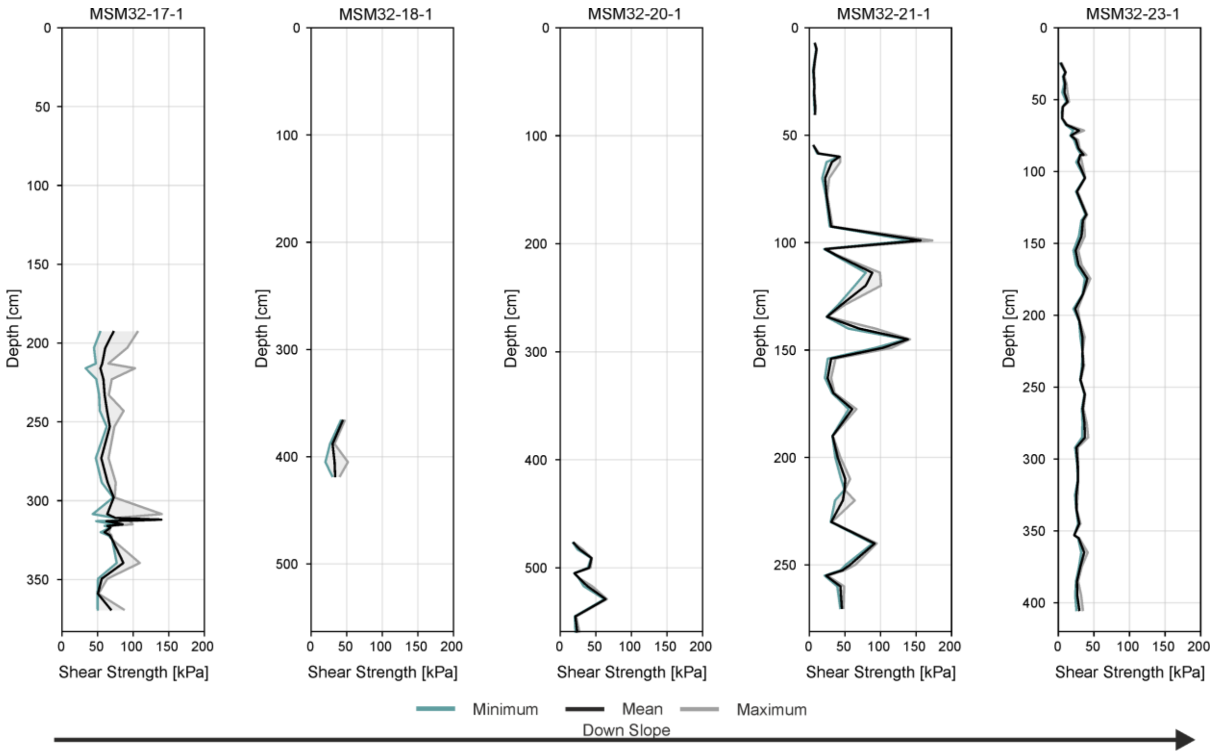

Figure S8. Plot showing the shear strength profiles of five sediment cores in downslope order *(see Fig. 1). The cores comprise remobilized sediments with destroyed consolidation profile compared to in-situ seafloor sediments (12).*

REFERENCES AND NOTES

1. G. Parker, Y. Fukushima, H. M. Pantin, Self-accelerating turbidity currents. *J. Fluid. Mech.* **171**, 145–181 (1986).

2. R. M. Iverson, The physics of debris flows. *Rev. Geophys.* **35**, 245–296 (1997).

3. S. W. McCoy, J. W. Kean, J. A. Coe, G. E. Tucker, D. M. Staley, T. A. Wasklewicz, Sediment entrainment by debris flows: In situ measurements from the headwaters of a steep catchment. *J. Geophys. Res.* **117**, F03016 (2012).

4. P. Gauer, M. Kern, K. Kristensen, K. Lied, L. Rammer, H. Schreiber, On pulsed Doppler radar measurements of avalanches and their implication to avalanche dynamics. *Cold Reg. Sci. Technol.* **50**, 55–71 (2007).

5. O. Hungr, S. McDougall, M. Bovis, "Entrainment of material by debris flows" in *Debris-Flow Hazards and Related Phenomena*, Springer Praxis Books (Springer, 2005), pp. 135–158.

6. D. Ericson, M. Ewing, B. C. Heezen, Turbidity currents and sediments in North Atlantic. *AAPG Bull.* **36**, 489–511 (1952).

7. W. R. Normark, Growth patterns of deep-sea fans. *AAPG Bull.* **54**, 2170–2195 (1970).

8. E. Mutti, *Turbidite Sandstones* (AGIP, Istituto di geologia, Università di Parma, 1992).

9. J. R. Curray, F. J. Emmel, D. G. Moore, The Bengal Fan: Morphology, geometry, stratigraphy, history and processes. *Mar. Petrol. Geol.* **19**, 1191–1223 (2002).

10. R. B. Wynn, P. P. E. Weaver, D. G. Masson, D. A. V. Stow, Turbidite depositional architecture across three interconnected deep-water basins on the north-west African margin. *Sedimentology* **49**, 669–695 (2002).

11. C. J. Stevenson, C. A. L. Jackson, D. M. Hodgson, S. M. Hubbard, J. T. Eggenhuisen, Deep-water sediment bypass. *J. Sediment. Res.* **85**, 1058–1081 (2015).

12. J. Peakall, J. Best, J. H. Baas, D. M. Hodgson, M. A. Clare, P. J. Talling, R. M. Dorrell, D. R. Lee, An integrated process-based model of flutes and tool marks in deep-water environments: Implications for palaeohydraulics, the Bouma sequence and hybrid event beds. *Sedimentology* **67**, 1601–1666 (2020).

13. P. J. Talling, J. Allin, D. A. Armitage, R. W. C. Arnott, M. J. B. Cartigny, M. A. Clare, F. Felletti, J. A. Covault, S. Girardclos, E. Hansen, P. R. Hill, R. N. Hiscott, A. J. Hogg, J. H. Clarke, Z. R. Jobe, G. Malgesini, A. Mozzato, H. Naruse, S. Parkinson, F. J. Peel, D. J. W. Piper, E. Pope, G. Postma, P. Rowley, A. Sguazzini, C. J. Stevenson, E. J. Sumner, Z. Sylvester, C. Watts, J. P. Xu, Key future directions for research on turbidity currents and their deposits. *J. Sediment. Res.* **85**, 153–169 (2015).

14. E. L. Pope, M. J. B. Cartigny, M. A. Clare, P. J. Talling, D. G. Lintern, A. Vellinga, S. Hage, S. Açikalin, L. Bailey, N. Chapplow, Y. Chen, J. T. Eggenhuisen, A. Hendry, C. J. Heerema, M. S. Heijnen, S. M. Hubbard, J. E. Hunt, C. McGhee, D. R. Parsons, S. M. Simmons, C. D. Stacey, D. Vendettuoli, First source-to-sink monitoring shows dense head controls sediment flux and runout in turbidity currents. *Sci. Adv.* **8**, eabj3220 (2022).

15. P. J. Talling, M. L. Baker, E. L. Pope, S. C. Ruffell, R. S. Jacinto, M. S. Heijnen, S. Hage, S. M. Simmons, M. Hasenhündl, C. J. Heerema, C. McGhee, R. Apprioual, A. Ferrant, M. J. B. Cartigny, D. R. Parsons, M. A. Clare, R. M. Tshimanga, M. A. Trigg, C. A. Cula, R. Faria, A. Gaillot, G. Bola, D. Wallance, A. Griffiths, R. Nunny, M. Urlaub, C. Peirce, R. Burnett, J. Neasham, R. J. Hilton, Longest sediment flows yet measured show how major rivers connect efficiently to deep sea. *Nat. Commun.* **13**, 4193 (2022).

16. J. E. Hughes-Clarke, A. N. Shor, D. J. W. Piper, L. A. Mayer, Large-scale current-induced erosion and deposition in the path of the 1929 grand banks turbidity current. *Sedimentology* **37**, 613–629 (1990).

17. C. J. Stevenson, P. J. Talling, D. G. Masson, E. J. Sumner, M. Frenz, R. B. Wynn, The spatial and temporal distribution of grain-size breaks in turbidites. *Sedimentology* **61**, 1120–1156 (2014).

18. R. B. Wynn, P. J. Talling, D. G. Masson, C. J. Stevenson, B. Cronin, T. L. Bas, D. C. Mosher, L. Moscardelli, C. Baxter, R. Urgeles, R. C. Shipp, J. D. Chaytor, H. J. Lee, Investigating the timing, processes and deposits of one of the world's largest submarine gravity flows: The 'bed 5 event' off Northwest Africa. *Adv. Nat. Tech. Haz. Res.* **28**, 463–474 (2010).

19. P. J. Talling, R. B. Wynn, D. G. Masson, M. Frenz, B. T. Cronin, R. Schiebel, A. M. Akhmetzhanov, S. Dallmeier-Tiessen, S. Benetti, P. P. E. Weaver, A. Georgiopoulou, C. Zühlsdorff, L. A. Amy, Onset of submarine debris flow deposition far from original giant landslide. *Nature* **450**, 541–544 (2007).

20. S. Krastel, R. B. Wynn, P. Feldens, A. Schürer, C. Böttner, C. Stevenson, M. J. B. Cartigny, V. Hühnerbach, D. Unverricht, Flow behaviour of a giant landslide and debris flow entering agadir canyon, NW Africa. *Adv. Nat. Tech. Haz. Res.* **41**, 145–154 (2016).

21. W. Li, S. Krastel, T. M. Alves, M. Urlaub, L. Mehringer, A. Schürer, P. Feldens, F. Cross, C. J. Stevenson, R. B. Wynn, The Agadir Slide offshore NW Africa: Morphology, emplacement dynamics, and potential contribution to the Moroccan Turbidite System. *Earth Planet. Sci. Lett.* **498**, 436–449 (2018).

22. J. E. Hunt, Identifying and quantifying erosion beneath the deposits of long-runout turbidity currents along their pathway. *Mar. Geol.* **389**, 32–51 (2017).

23. J. E. Hunt, R. B. Wynn, P. J. Talling, D. G. Masson, Frequency and timing of landslide-triggered turbidity currents within the Agadir Basin, offshore NW Africa: Are there associations with climate change, sea level change and slope sedimentation rates? *Mar. Geol.* **346**, 274–291 (2013).

24. J. J. Mountjoy, J. D. Howarth, A. R. Orpin, P. M. Barnes, D. A. Bowden, A. A. Rowden, A. C. G. Schimel, C. Holden, H. J. Horgan, S. D. Nodder, J. R. Patton, G. Lamarche, M. Gerstenberger, A. Micallef, A. Pallentin, T. Kane, Earthquakes drive large-scale submarine canyon development and sediment supply to deep-ocean basins. *Sci. Adv.* **4**, eaar3748 (2018).

25. H. A. Macdonald, R. B. Wynn, V. A. Huvenne, J. Peakall, D. G. Masson, P. P. Weaver, S. D. McPhail, New insights into the morphology, fill, and remarkable longevity (>0.2 m.y.) of modern deep-water erosional scours along the northeast Atlantic margin. *Geosphere* **7**, 845–867 (2011).

26. D. J. W. Piper, A. E. Aksu, The source and origin of the 1929 Grand Banks turbidity-current inferred from sediment budgets. *Geo-Mar. Lett.* **7**, 177–182 (1987).

27. C. J. Heerema, P. J. Talling, M. J. Cartigny, C. K. Paull, L. Bailey, S. M. Simmons, D. R. Parsons, M. A. Clare, R. Gwiazda, E. Lundsten, K. Anderson, K. L. Maier, J. P. Xu, E. J. Sumner, K. Rosenberger, J.

Gales, M. McGann, L. Carter, E. Pope, What determines the downstream evolution of turbidity currents? *Earth Planet. Sci. Lett.* **532**, 116023 (2020).

28. W. O. Symons, E. J. Sumner, C. K. Paull, M. J. B. Cartigny, J. P. Xu, K. L. Maier, T. D. Lorenson, P. J. Talling, A new model for turbidity current behavior based on integration of flow monitoring and precision coring in a submarine canyon. *Geology* **45**, 367–370 (2017).

29. J. P. Xu, O. E. Sequeiros, M. A. Noble, Sediment concentrations, flow conditions, and downstream evolution of two turbidity currents, Monterey Canyon, USA. *Deep-Sea Res. Pt.* **89**, 11–34 (2014).

30. M. Azpiroz-Zabala, M. J. B. Cartigny, P. J. Talling, D. R. Parsons, E. J. Sumner, M. A. Clare, S. M. Simmons, C. Cooper, E. L. Pope, Newly recognized turbidity current structure can explain prolonged flushing of submarine canyons. *Sci. Adv.* **3**, e1700200 (2017).

31. C. K. Paull, P. J. Talling, K. L. Maier, D. Parsons, J. P. Xu, D. W. Caress, R. Gwiazda, E. M. Lundsten, K. Anderson, J. P. Barry, M. Chaffey, T. O'Reilly, K. J. Rosenberger, J. A. Gales, B. Kieft, M. McGann, S. M. Simmons, M. McCann, E. J. Sumner, M. A. Clare, M. J. Cartigny, Powerful turbidity currents driven by dense basal layers. *Nat. Commun.* **9**, 4114 (2018).

32. S. K. Hsu, J. Kuo, C. L. Lo, C. H. Tsai, W. B. Doo, C. Y. Ku, J. C. Sibuet, Turbidity currents, submarine landslides and the 2006 Pingtung earthquake off SW Taiwan. *Terr. Atmos. Ocean Sci.* **19**, 767–772 (2008).

33. M. A. Clare, I. A. Yeo, S. Watson, R. Wysoczanski, S. Seabrook, K. Mackay, J. E. Hunt, E. Lane, P. J. Talling, E. Pope, S. Cronin, M. Ribó, T. Kula, D. Tappin, S. Henrys, C. de Ronde, M. Urlaub, S. Kutterolf, S. Fonua, S. Panuve, D. Veverka, R. Rapp, V. Kamalov, M. Williams, Fast and destructive density currents created by ocean-entering volcanic eruptions. *Science* **381**, 1085–1092 (2023).

34. L. Carter, J. D. Milliman, P. J. Talling, R. Gavey, R. B. Wynn, Near-synchronous and delayed initiation of long run-out submarine sediment flows from a record-breaking river flood, offshore Taiwan. *Geophys. Res. Lett.* **39**, L12603 (2012).

35. C. L. Amos, D. C. Mosher, Erosion and deposition of fine-grained sediments from the bay of fundy. *Sedimentology* **32**, 815–832 (1985).

36. J. Ikeda, F. Y. Testik, Propagation, deposition, and suspension characteristics of constant-volume particle-driven gravity currents. *Environ. Fluid Mech.* **21**, 177–208 (2021).

37. C. Gladstone, J. Phillips, R. Sparks, Experiments on bidisperse, constant-volume gravity currents: Propagation and sediment deposition. *Sedimentology* **45**, 833–843 (1998).

38. J. H. Baas, J. L. Best, J. Peakall, M. Wang, A phase diagram for turbulent, transitional, and laminar clay suspension flows. *J. Sediment. Res.* **79**, 162–183 (2009).

39. J. H. Baas, J. L. Best, J. Peakall, Depositional processes, bedform development and hybrid bed formation in rapidly decelerated cohesive (mud–sand) sediment flows. *Sedimentology* **58**, 1953–1987 (2011).

40. P. J. Talling, D. G. Masson, E. J. Sumner, G. Malgesini, Subaqueous sediment density flows: Depositional processes and deposit types. *Sedimentology* **59**, 1937–2003 (2012).

41. W. H. McAnally, C. Friedrichs, D. Hamilton, E. Hayter, P. Shrestha, H. Rodriguez, A. Sheremet, A. Teeter, ASCE Task Committee on Management of Fluid Mud, Management of fluid mud in estuaries, bays, and lakes. I: Present state of understanding on character and behavior. *J. Hydraul. Eng.* **133**, 9–22 (2007).

42. D. W. Caress, D. N. Chayes, "New software for processing sidescan data from sidescan-capable multibeam sonars" in *Oceans '95 Mts/Ieee - Challenges of Our Changing Global Environment, Conference Proceedings*, (IEEE, 1995), vol. 2, pp. 997–1000.

43. J. D. Hunter, Matplotlib: A 2D graphics environment. *Comput. Sci. Eng.* **9**, 90–95 (2007).

44. W. McKinney, pandas: A foundational Python library for data analysis and statistics. *Python High Perform. Sci. Comput.* **14**, 1–9 (2011).

45. C. R. Harris, K. J. Millman, S. J. van der Walt, R. Gommers, P. Virtanen, D. Cournapeau, E. Wieser, J. Taylor, S. Berg, N. J. Smith, R. Kern, M. Picus, S. Hoyer, M. H. van Kerkwijk, M. Brett, A. Haldane, J. F. del Río, M. Wiebe, P. Peterson, P. Gérard-Marchant, K. Sheppard, T. Reddy, W. Weckesser, H. Abbasi, C. Gohlke, T. E. Oliphant, Array programming with NumPy. *Nature* **585**, 357–362 (2020).

46. M. Stuiver, P. J. Reimer, Extended ^{14}C data base and revised calib 3.0 ^{14}C age calibration program. *Radiocarbon* **35**, 215–230 (1993).

47. T. J. Heaton, P. Köhler, M. Butzin, E. Bard, R. W. Reimer, W. E. N. Austin, C. B. Ramsey, P. M. Grootes, K. A. Hughen, B. Kromer, P. J. Reimer, J. Adkins, A. Burke, M. S. Cook, J. Olsen, L. C. Skinner, Marine20—The marine radiocarbon age calibration curve (0-55,000 Cal Bp). *Radiocarbon* **62**, 779–820 (2020).

48. R. B. Wynn, B. T. Cronin, J. Peakall, Sinuous deep-water channels: Genesis, geometry and architecture. *Mar. Petrol. Geol.* **24**, 341–387 (2007).

49. R. Soulsby, *Dynamics of Marine Sands* (Thomas Telford, 1997).

50. H. Kuhlmann, H. Meggers, T. Freudenthal, G. Wefer, The transition of the monsoonal and the N Atlantic climate system off NW Africa during the Holocene. *Geophys. Res. Lett.* **31**, L22204 (2004).

51. A. Hurst, A. Scott, M. Vigorito, Physical characteristics of sand injectites. *Earth Sci. Rev.* **106**, 215–246 (2011).

52. J. A. Ross, J. Peakall, G. M. Keevil, An integrated model of extrusive sand injectites in cohesionless sediments. *Sedimentology* **58**, 1693–1715 (2011).
